# Birds of a Feather can Butt Heads: When Machiavellian Employees Work with Machiavellian Leaders

**DOI:** 10.1007/s10551-016-3251-2

**Published:** 2016-07-05

**Authors:** Frank D. Belschak, Rabiah S. Muhammad, Deanne N. Den Hartog

**Affiliations:** 1 Amsterdam Business School, University of Amsterdam, Plantage Muidergracht 12, 1018 TV Amsterdam, The Netherlands; 2 Department of Psychology, University of Maryland, College Park, MD 20742 USA

**Keywords:** Leader–follower fit, Machiavellianism, Machiavellian leadership, Counterproductive work behavior, Stress, Trust, Unethical behavior

## Abstract

Machiavellians are manipulative and deceitful individuals willing to utilize any strategy or behavior needed to attain their goals. This study explores what occurs when Machiavellian employees have a Machiavellian leader with the same negative, manipulative disposition. We argue that Machiavellian employees have a negative worldview and are likely to trust their leaders less. This reduced trust likely results in these employees experiencing higher stress and engaging in more unethical behavior. In addition, we expect these negative relationships to be exacerbated when such followers experience Machiavellian leadership. Thus, we test a moderated mediation model assessing whether Machiavellianism affects employees and whether combining Machiavellian leaders and Machiavellian employees is toxic in the sense of exacerbating the negative impact of Machiavellianism on employee trust. Results do not support the proposed conditional indirect effect of trust for either stress or unethical behavior. Instead, we find a conditional direct effect of employee Machiavellianism on both trust and stress: When Machiavellian employees have Machiavellian leaders, their trust in their leader significantly decreases, and their level of stress significantly increases. We also find support for an unconditional indirect effect of trust for employee stress (but not for unethical work behaviors), Machiavellianism in employees relates to stress via lowered trust in the leader. For unethical behavior, we only find a main effect of employee Machiavellianism.

## Introduction

Numerous studies have characterized Machiavellianism (Mach) as a ‘dark’ personality trait (see Furnham et al. 2013) and have argued that Machiavellians threaten the well-being of the organization and its members (see Dahling et al. 2009, 2012). Both employee Mach and leader Mach have been linked to manipulative, unethical, and counterproductive work behaviors (e.g., Dahling et al. 2009; Kiazad et al. 2010). But what happens if *both* leader and employee score high on a negative personality trait such as Machiavellianism? Does this exacerbate the problem? Does similarity between leader and employee on the Machiavellianism trait lead to positive outcomes as leader–follower fit theory might suggest (e.g., Atwater and Dionne 2007)? Or do such negative personalities clash, resulting in negative outcomes? The effects of the interaction between negative leader and follower traits have hardly been investigated to date. Therefore, the aim of this paper was to explore how employees who score high on Machiavellianism contend with having a similarly manipulative high-Mach leader.

The construct of Machiavellianism is named after the Italian Renaissance diplomat Niccolo Machiavelli who described in his thesis the ideal but unethical behavior of royalty to successfully achieve their goals, but it was not until the work of Christie and Geis (1970) that Mach was introduced as a psychological construct. According to these authors, Mach describes an individual who is a master manipulator, someone who uses aggressive tactics, acts amorally, and has an untrusting, negative, and cynical view of the world. Due to its manipulative and amoral side, Mach is usually described in a negative light and has attracted attention in work on organizational behavior (e.g., Belschak et al. 2015; Dahling et al. 2009, 2012) as well as business ethics (e.g., Den Hartog and Belschak 2012; Ricks and Fraedrich 1999; Schepers 2003). Machs are convincing liars and manipulators, who are less sensitive to ethical issues (e.g., Schepers 2003) and are found in any type of organization, even charitable organizations (Smith et al. 2009; Chen 2010). Thus, understanding how they impact others and organizations is important, and recent research has focused on how such “dark” personality traits in organizational members affect different types of organizational outcomes (Harms et al. 2011; Judge et al. 2009; Kuyumcu and Dahling 2014).

A defining characteristic of Machiavellians is their cynical worldview (see Jones and Paulhus 2009). Machiavellians expect the worst from others, assume that others are cheating and lying, and thus distrust others and their motives (e.g., Dahling et al. 2009). This negative worldview and the resulting lack of trust in others around them may explain Machiavellians’ increased level of negative feelings (stress, dissatisfaction) (e.g., Dahling et al. 2009) and their manipulative and amoral behavior (e.g., ‘strike before the other does’) (e.g., Mudrack 1993; Schepers 2003). Machiavellian employees are more likely not to trust others, including their leaders, and research has shown that a lack of trust between leader and follower has a negative impact on follower attitudes and behaviors (see Dirks and Ferrin 2002). We therefore argue that lowered trust in the leader mediates the relationship between employee Mach and employee well-being (stress) and (unethical, counterproductive) work behaviors.

The extant literature on Mach has mainly focused on Mach employees (e.g., Belschak et al. 2015; Dahling et al. 2009) or (to a lesser extent) on Mach leaders (e.g., Deluga 2001; Den Hartog and Belschak 2012). As noted, what is currently missing in the literature is an understanding of whether reactions of Machs may alter when they interact with other Machs, in particular Mach employees with Mach leaders. The idea that high-Mach employees will react differently to Mach leaders than low-Mach employees can be theoretically linked to leader–follower fit theory (e.g., Atwater and Dionne 2007) which argues that leader and follower should be congruent in terms of personal values or personality. While a positive impact of similar personalities is likely for some traits, such as conscientiousness (e.g., Antonioni and Park 2001), for other characteristics, interactions between dissimilar personalities are more advantageous (e.g., Grant et al. 2011; Kristof-Brown et al. 2005).

**Fig. 1:**
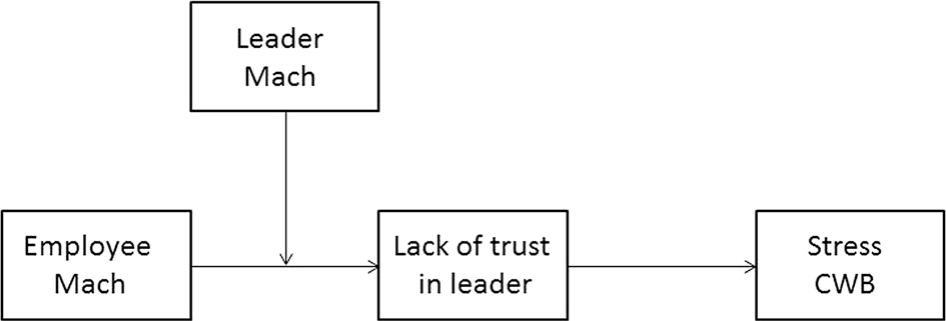
A moderated mediation model of employee–leader Machiavellianism

Here, we posit that Machiavellianism is a trait for which similarity between leader and employee yields particularly negative outcomes, and the combination of a Mach employee with a Mach leader likely forms a toxic combination. Hence, we propose a moderated mediation model in which Machiavellian followers are likely to exhibit low trust which, in turn, relates to having more stress and engaging in more counterproductive work behaviors (CWB), and this low trust is exacerbated when they have a Mach leader who acts similarly manipulatively and whom they cannot control. Our model is summarized in Fig. 1.

This paper contributes to the literature in several respects. First, we add to the literature on Machiavellianism and the wider fields of business ethics and unethical behavior (e.g., Belschak et al. 2015; Dahling et al. 2009, 2012) by investigating the effects of the potentially destructive combination of Mach leaders with Mach employees. Second, we add to the literature on leader–follower fit and leader–follower value congruence (e.g., Atwater and Dionne 2007; Meglino et al. 1989) by investigating the contingencies under which such personality fit or congruence leads to undesirable outcomes for the individual and the organization. Finally, we contribute to the broader leadership literature by exploring the (moderating) effects of leader personality on follower behaviors (e.g., Judge et al. 2002) and addressing Machiavellian leadership as another negative and potentially destructive type of leadership.

### Machiavellian Employees

Researchers have noted that employees’ negative personality traits can negatively affect employee work behaviors (see Furnham et al. 2013; Wu and Lebreton 2011). For instance, employee narcissism is a stable and strong predictor of CWB (e.g., Penney and Spector 2002). In this vein, Christie and Geis (1970) already argued that Machiavellians possess an unethical attitude, display a lack of affect in interpersonal relationships, perceive of others solely as tools, and are strongly goal-oriented but shortsighted beyond their immediate goals. Compared to those low in Mach, employees who are high in Mach are more manipulative, win more, are more persuasive (Christie and Geis 1970; Schepers 2003), have a higher internal locus of control (Gable and Dangello 1994), and engage in more emotional blackmail (Chen 2010).

Geis and Moon (1981) found that high Machs are very convincing liars. Smith et al. (2009) investigated non-profit organization employees’ tendencies toward Mach and discovered that even within the realm of supporting others, employees high in Mach had a propensity for unethical behavior (using donor gift for unintended purposes, etc.). Research has also shown that being high in Mach can influence the decision-making process related to ethical judgments, such that Machs are less likely to perceive ethical issues (Schepers 2003). These findings suggest that employees who are high in Mach are more likely to exhibit behaviors that are unethical and harmful to others around them and to the organization (see Dahling et al. 2009, 2012 for an overview on Mach and organizational criteria).

An evolutionary psychological perspective similarly suggests that Machs are more willing to make unethical choices and to engage in destructive behaviors (Wilson et al. 1998). Because of their manipulative skills and lack of feelings of guilt, Machs should be best served by secretly exploiting an organization while hiding their true nature as long as possible and changing organizations when discovered (Wilson et al. 1996). Machs therefore have a ‘natural’ tendency to engage in exploitative and counterproductive work behaviors (e.g., Dahling et al. 2009; Nelson and Gilbertson 1991). In line with this argument, high Machs show a general propensity to engage in antisocial behavior (see Jones and Paulhus 2009), and game theoretical experiments found that high Machs chose economic opportunism and maximization of their own profits rather than cooperation (Gunnthorsdottir et al. 2002; Sakalaki et al. 2007).

Employee Mach is not only linked to negative work behaviors, employees high on Mach also experience negative work attitudes and emotions. Mach employees are consistently dissatisfied with the status quo and are constantly in search of ways to gain more influence over others (Fehr et al. 1992), which results in highly stressful feelings of pressure (Dahling et al. 2009). Stress has been defined as a person’s perception that job factors (over)challenge his/her abilities and resources, and it can change his or her psychological and/or physiological state to the extent that the person does not function normally (Newman and Beehr 1979). The consequences of workplace stress include job stagnation, burnout, poor performance, and less effective interpersonal relations (Manshor et al. 2003). One of the prominent ways of relieving stress includes developing and sustaining a strong social support system (Fenlason and Beehr 1994). For employees who are high on Mach, this may be problematic. They likely tend to experience high stress and pressure, yet are less likely to benefit from social support as Machs view the world through a pessimistic lens, are highly cynical of others, and distrust others’ motives (Dahling et al. 2009). Consistently, previous research demonstrates that high Mach is positively related to workplace stress (Gable and Topol 1991; Gemmill and Heisler 1972; Heisler and Gemmill 1977).

### A Mediation Model of Employee Machiavellianism

Machiavellians’ negative work behaviors and emotions may be related to their distrusting nature (Christie and Geis 1970; Gurtman 1992). Cook and Wall (1980, p. 39) define trust as “the extent to which one is willing to ascribe good intentions to and have confidence in the words and actions of other(s)…this willingness will in turn affect the way in which one behaves towards others.” Trust is an essential component in maintaining a healthy social exchange relationship with others (Blau 1964). It can increase information sharing and cooperation (Solomon and Flores 2001; McAllister 1995), relate to performance (Dirks 2000), and reduce job stress (Vigoda-Gadot and Talmud 2010).

Individuals who are high in Mach display a negative and cynical worldview (Christie and Geis 1970; Jones and Paulhus 2009), and this negative worldview impacts their ability to positively evaluate others as worthy or safe to display vulnerability toward. This can lead to a lack of trust in others (Gurtman 1992; Higgins and King 1981). Consistently, Gurtman (1992) found that those who were high in Mach had interpersonal problems relating to a lack of trust (see also, Horowitz et al. 1988). Similarly, Dahling et al. (2009) propose that a lack of trust is a key characteristic of Machs. Since Machs base their perceptions of others on how they themselves would act, it is difficult for them to trust others. As Machs constantly manipulate and attempt to take advantage of others, they assume that others are also trying to control and manipulate them, and thus they will tend not to trust others.

Employee trust is closely linked to many important employee work attitudes and behaviors. For example, having low trust in the leader likely results in employees showing less cooperative behavior and experiencing more stress. Dirks and Ferrin (2002) provide a review and meta-analysis on the relationship between employee trust and organizational outcomes (e.g., job (dis)satisfaction and extra-role work behaviors). Employees who do not trust their leaders are likely to experience more frustration, negative emotions, and conflict. Also, employees often see the support by and characteristics of their leader as indicative of their organization’s support and characteristics (e.g., Eisenberger et al. 1986; Rhoades et al. 2001). Based on exchange theory (e.g., Blau 1964), scholars therefore argue that employees reciprocate their leader for being trustworthy by showing positive work attitudes and pro-organizational behaviors such as organizational citizenship behavior (e.g., Konovsky and Pugh 1994; Yang et al. 2009). In contrast, Machiavellian employees who do not trust their leader are likely not to invest in positive behaviors and rather engage in selfish or unethical behaviors instead (e.g., not work hard when things need to be done, steal work material). Consistently, research has shown that employees who are frustrated and experience negative emotions and conflict are more likely to engage in counterproductive work behaviors (e.g., Fox and Spector 1999; Fox et al. 2001). Employees do not necessarily express such aggressive behaviors immediately to the source of their negative experiences (e.g., their leader) but may also engage in displaced aggressive acts toward other targets (e.g., coworkers, or the organization) (e.g., Mitchell and Ambrose 2012). Researchers have noted that such displaced aggressive acts are a common reaction (Marcus-Newhall et al. 2000).

Research has further shown that a lack of trust is often related to interpersonal problems and related stress (e.g., Rotter 1980). As leaders have the authority to take decisions that have the potential to substantially (negatively) affect employees, employees who do not trust their leaders are likely to invest time and effort in ‘covering their backs’ which is usually experienced as stressful (e.g., Mayer and Gavin 2005). Also, a lack of trust in one’s leader can lead to feelings of powerlessness and helplessness in employees that are experienced as stressful (e.g., Gillespie et al. 2001). Prior research has shown that cynical personalities like Mach score low on trust in others (e.g., seeing others as dishonest and unreliable) and that such low levels of trust are linked to high levels of interpersonal stress (e.g., Greenglass and Julkunen 1989; Gurtman 1992). Based on the theoretical arguments provided above, we thus hypothesize:

#### **Hypothesis 1**

Low trust in the leader mediates the positive relationship between employee Mach and employee counterproductive behavior (Hypothesis 1a) and between employee Mach and employee stress (Hypothesis 1b).

### The Moderating Role of Leader Machiavellianism

While research on Mach in leaders is still scarce, interesting findings have come to light about the impact of Mach leaders on others. Mach leaders have been found to be adaptable to situations, but detached from their employees’ interpersonal concerns (Dahling et al. 2009; Deluga 2001; Drory and Gluskinos 1980). These leaders are focused on organizational politics and seek to control employees (McHoskey 1999). The topic most investigated in regard to *Machiavellian leadership* (i.e., leadership behavior of leaders scoring high on the trait of Mach) is the extensive use of impression management to manipulate employees for personal gain (Becker and O’Hair 2007). Mach leaders are skilled at creating a desirable image, and Mach was found to be positively related to charismatic leadership (Deluga 2001). Yet, studies also show that generally Machs rely on deceptive strategies and lying in social relationships (e.g., DePaulo and Rosenthal 1979; Geis and Moon 1981; Gunnthorsdottir et al. 2002). Their persuasive powers are such that they can influence others in ways that run counter to organizational goals and individuals’ own pro-social values (Bolino and Turnley 2003). Machiavellians show a strong goal focus and a lack of feelings of guilt and emotional concerns regarding how to achieve these goals (Christie and Geis 1970; Cooper and Peterson 1980), and we expect that Mach leaders thus put pressure on their subordinates to meet their targets no matter how. In support, Kiazad et al. (2010) found that supervisors who rated themselves as high on Mach were also more often perceived to be abusive.

Here, we propose that high-Mach employees will react even more strongly negatively to having a leader who is similarly high on Mach. As noted, the idea that high-Mach employees will react differently to high-Mach leaders than low-Mach employees can be theoretically linked to leader–follower fit theory (e.g., Atwater and Dionne 2007). This theory stresses the development of a productive relationship between leaders and employees as a means of effective job fulfillment (Tjosvold 1985). While fit has traditionally been examined at the organizational or group level, Atwater and Dionne (2007) theoretically developed the idea of fit within the dyad between leaders and their employees. Leader–follower fit focuses on the level of similarity within the dyad and is primarily concerned with the relevance of similarity in values, personality, and beliefs in the formation of these relationships and the impact on important outcomes (Atwater and Dionne 2007; Heilman 1983). Similarly attraction–selection–attrition theory (Schneider 1987) also predicts that those who share similar features or values within an organization are likely to experience rewarding interactions and be attracted to each other.

While similar personalities and values in most cases should create a common ground or platform between the leader and the employee (Bauer and Green 1996; Senger 1971; Turban and Jones 1988), this is likely to be different for Mach as a trait. Here, we would predict that a high fit does not lead to positive but to negative outcomes. Similar to the idea of fitting puzzle pieces, for some traits, when a leader displays one type of personality, the ideal employee match is their opposite in personality. This helps to prevent negative interactions and reduced performance. Thus, although for some characteristics similarity may be optimal, for others complementarity has more positive effects (see, e.g., dominance complementarity theory, predicting a better fit between leader dominance and employee submission (or vice versa) than between leader dominance and employee dominance, see Grant et al. 2011).

We propose that Mach is a trait for which both leader and employee being high exacerbates negative effects, and a match of high-Mach personalities between leader and employee is likely to form a toxic combination that is especially disadvantageous for the employee. Instead of building a positive empowering relationship based on positive similarity, Mach leaders with Mach employees are likely to collide and interact in destructive ways because both the Mach leader and the Mach employee are trying to control and manipulate each other. Previous research has shown that when two dominant personalities clash, the one who is higher in the hierarchy and has more power has the upper hand (Haythorn and Altman 1967; Jehn 1995, 1997), thus we expect that this clash between Mach leaders and employees is especially disadvantageous for the employee.

Because Machiavellians view the world negatively and ascribe bad intentions to others (Christie and Geis 1970), Mach employees find it hard to trust others around them. Gunnthorsdottir et al. (2002) found that as a result of their low trust in relationship partners, Machs were significantly less likely to reciprocate during a bargaining game and were the least likely to extend trust first. Such lack of trust should even be worse when working with a Mach leader whom Mach employees likely come to perceive as being even more manipulative, deceitful, and exploitative than a non-Mach leader. In particular, in case of a Mach leader, Mach employees’ expectations of what they want (e.g., being in control and having the freedom to act the way they want) and what they receive from their Mach leader (tight monitoring, a wary and distrusting leader) may be too disparate for the development of a trusting or healthy relationship. Thus, for Mach employees trust is already a scarce resource (Dahling et al. 2009; Gurtman 1992), combined with a leader they cannot manipulate (and whom they suspect to manipulate them), and who is likely to similarly signal a low level of trust in employees, we predict that Mach employees trust Mach leaders significantly less than non-Mach leaders. Combined with Hypothesis 1, we therefore propose a moderated mediation model hypothesis:

#### **Hypothesis 2:**

Leader Mach will moderate the strength of the relationship between employee Mach and employee CWB (Hypothesis 2a) and stress (Hypothesis 2b) via employee trust, such that the mediated relationship will be stronger under leaders high in Mach than under leaders low in Mach.

## Method

### Sample and Procedure

To test our hypotheses, we conducted a multi-source field study among managers and their employees in the Netherlands. We aimed for a diverse sample of employees from different industries and professional backgrounds to increase the representativeness of the study. Participating companies came from a variety of industries ranging from banking to retailing, and from IT to governmental organizations. Managers and (via the managers) employees were contacted and asked for their cooperation in a study on leadership. Only employee questionnaires with a matching manager survey that were completely filled out were included in the analyses. Responses were sent directly to the researchers, who were available to answer questions. In total 350 employee–supervisor dyads were approached of which one hundred and 96 matched employees and managers (i.e., 196 complete dyads) completed the surveys and were included in the study (response rate of 56 %).

Participation in our study was voluntary and anonymous, and participants did not receive anything in return. Respondents (employees) worked in a wide range of jobs including administration, salespeople, customer service employees, consultants, and clerks. 54 % of the focal employees were men; the average age of the respondents was 31.3 years (*SD* = 11.4), and they had worked for their current employer for 5.2 years on average (*SD* = 7.6). Managers knew the employees they rated for 3.3 years on average (*SD* = 3.5).

### Measures

Responses for all items were given on a seven-point scale (1 = ‘completely disagree’ to 7 = ‘completely agree’); all items were administered in Dutch. The survey of the employee included measures of Machiavellianism, trust, stress, and unethical behavior (CWB); the survey of the managers included a measure of Machiavellianism.

*Machiavellianism* both in the employee and the manager survey was measured by 8 items from the Mach-IV scale from Christie and Geis (1970). Examples are “It is wise to flatter important people” and “Never tell anyone the real reason you did something unless it is useful to do so.” This is the most widely used measure of Machiavellianism (e.g., Deluga 2001; Paulhus and Williams 2002), and specifically the eight-item version of the Mach-IV was successfully used in other studies in the Netherlands before (e.g., Belschak et al. 2015; Den Hartog and Belschak 2012). Cronbach’s alpha was .84 for both the employee version and the manager version of the measure.

*Trust* *in the leader* was measured by three items (e.g., “My manager can be trusted to make sensible decisions”) adapted from Cook and Wall (1980). Earlier studies have similarly used short subscales of this measure translated in Dutch (e.g., Kalshoven et al. 2011). Cronbach’s was .82.

The measure of employees’ *experienced stress* consisted of three items (we excluded the one reverse-coded item of the original scale due to translation issues) from Motowidlo et al. (1986) (e.g., “I feel a great deal of stress because of my work”). Cronbach’s alpha was .85.

*Counterproductive work behaviors* were measured with the minor organizational CWB subscale from Fox and Spector (1999) consisting of 11 items. This scale has been successfully used with Dutch samples before (e.g., Den Hartog and Belschak 2012). Sample items are “I purposely wasted company materials/supplies” and “I stayed home from work and said I was sick when I was not.” Cronbach’s alpha was .87.

We included *employee age and gender* and *leader gender* and *number of years that the leader was supervising this specific employee* and assessed whether they needed to be included as control variables in our analyses (see below).

## Results

To test the factor structure and the convergent and discriminatory validity of our scales, we conducted a confirmatory factor analysis (CFA) of the measures. To achieve a satisfactory indicator to sample ratio (which should be at least 1:5; see, e.g., Bentler and Chou 1987; Bentler 1995) we used a parceling approach (e.g., Bagozzi and Heatherton 1994). More specifically, we created three parcels of indicators for each of the longer and well-validated scales (i.e., three parcels for employee Mach, three parcels for leader Mach, and three parcels for employee CWB). For building parcels we followed a factorial algorithm as described by Rogers and Schmitt (2004) and Little et al. (2002). More specifically, we conducted an initial factor analysis for each construct to be parceled and built parcels according to the factor loadings of the items. Such an approach seemed adequate as the Mach-IV as well as our measure of CWB have been treated as unidimensional in the extant literature, and the results of our initial factor analyses supported this unidimensionality. As the stress and trust scales consisted only of three items each we did not form parcels but rather kept single items.

The CFA showed a satisfactory fit of the hypothesized five-factor structure (i.e., employee Mach, leader Mach, employee trust, stress, and CWB): *χ* ^2^ (80) = 168.93 (n.s.); *CFI* = .94; *IFI* = .94; *RMSEA* = *RMR* = .08. Factor loadings were significant and ranged from .70 to .90 for employee Mach, from .78 to .83 for leader Mach, from .76 to .80 for employee trust, from .73 to .94 for employee stress, and from .75 to .82 for employee CWB. Factor intercorrelations ranged from −.35 (employee Mach with employee trust) to .60 (employee Mach with employee CWB).

Table 1 presents the descriptives and intercorrelations of the scales. Both employee Mach and leader Mach were significantly related to employee trust, stress, and CWB, although the leader Mach–employee stress relationship was borderline with *p* = .052.

**Table 1:** Descriptives and inter-correlations of variables (Cronbach’s Alpha on diagonal)

	Mean	SD	Employee Mach	Leader Mach	Trust	Stress	CWB	Age	Gender	Leader gender	Years leading
Employee Mach	3.08	1.09	(.84)								
Leader Mach	2.89	1.09	.48**	(.84)							
Trust	5.43	1.10	−.28**	−.28**	(.82)						
Stress	3.41	1.28	.15*	.14	−.18*	(.85)					
CWB	1.91	.78	.46**	.33**	−.25**	.13	(.87)				
Employee age	31.34	11.43	−.07	.02	−.15*	.16*	.09	–			
Employee gender	1.46	.50	−.13	−.02	−.01	−.06	−.07	−.13	–		
Leader gender	1.33	.47	−.06	−.13	.02	.01	.01	−.04	.29**	–	
Years leading	3.30	3.46	.08	.14	−.06	.11	.23**	.35**	−.01	−.05	–

*N* = 196

* *p* < .05; ** *p* < . 01

To test our hypotheses, we used a regression-based approach to conducting moderated mediation analysis that involves bootstrapping to calculate 95 % bootstrap confidence intervals (e.g., Preacher et al. 2007; Hayes 2013). More specifically, we tested a moderated mediation model using the PROCESS macro (version 2.13.2; SPSS version 22) developed by Hayes (2013). We used the PROCESS option to center the predictors around their respective means and based the interaction term on these mean-centered scores. First, we conducted the analyses including all control variables. Next, we recalculated the analyses including only the control variables that were significantly correlated with the dependent variables in the correlations (see Table 1) (as suggested by Becker 2005). Finally, we reran the analyses without the control variables. Results of the three different computations were almost identical, and the significance levels of the results as well as the confidence intervals remained mainly unchanged. In line with suggestions of more recent literature on the treatment of control variables (e.g., Bernerth and Aguinis 2016; Spector and Brannick 2011) we therefore present the results of the analyses without control variables below.

The index of moderated mediation shows that the hypothesized moderated mediation model is not supported (stress: Index = .02, boot s.e. = .02, 95 % CI [−.00, .06]; CWB: Index = .01, boot s.e. = .01, 95 % CI [−.00, .04]). The regression coefficients are presented in Table 2; Table 3 provides the results of the tests regarding the conditional indirect and direct effects.

**Table 2:** Results of moderated mediation analysis using PROCESS (unstandardized coefficients, *N* = 196)

Employee trust *R* ^2^ = .14	B	s.e.	*t*	*p*	LLCI	ULCI
Employee Mach	−.17	.08	−2.24	.03	−.33	−.02
Leader Mach	−.14	.08	−1.74	.08	−.30	.02
Employee Mach × leader Mach	−.15	.06	−2.71	.01	−.27	−.04

Employee stress *R* ^2^ = .07	B	s.e.	*t*	*p*	LLCI	ULCI
Employee Mach	.09	.10	.95	.35	−.10	.28
Leader Mach	.03	.10	.27	.79	−.17	.22
Employee Mach × leader Mach	.15	.07	2.16	.03	.01	.29
Employee trust	−.12	.09	−1.41	.16	−.30	.05

Employee CWB *R* ^2^ = .24	B	s.e.	*t*	*p*	LLCI	ULCI
Employee Mach	.27	.05	5.09	.00	.16	.37
Leader Mach	.09	.05	1.78	.08	−.01	.20
Employee Mach × leader Mach	−.02	.04	−.46	.65	−.09	.06
Employee trust	−.08	.05	−1.67	.10	−.18	.01

**Table 3:** Results of moderation analyses using PROCESS (*N* = 196)

	Effect	Boot s.e.	Boot LLCI	Boot ULCI
*Conditional indirect effect of employee Mach on stress via trust*				
Low leader Mach	.00	.02	−.03	.05
Mean leader Mach	.02	.02	−.00	.09
High leader Mach	.04	.03	−.01	.13

	Effect (s.e.)	*t* (*p*)	LLCI	ULCI
*Conditional direct effect of employee Mach on stress*				
Low leader Mach	−.07 (.13)	−.60 (.55)	−.32	.17
Mean leader Mach	.09 (.10)	.95 (.35)	−.10	.28
High leader Mach	.25 (.12)	2.15 (.03)	.02	.49

	Effect (s.e.)	*t* (*p*)	LLCI	ULCI
*Conditional direct effect of employee Mach on trust*				
Low leader Mach	−.01 (.10)	−.06 (.95)	−.21	.20
Mean leader Mach	−.17 (.08)	−2.24 (.03)	−.33	−.02
High leader Mach	−.34 (.09)	−3.61 (.00)	−.53	−.15

	Effect	Boot s.e.	Boot LLCI	Boot ULCI
*Conditional indirect effect of employee Mach on CWB via trust*				
Low leader Mach	.00	.01	−.02	.03
Mean leader Mach	.01	.01	−.00	.06
High leader Mach	.03	.02	−.00	.10

	Effect (s.e.)	*t* (*p*)	LLCI	ULCI
*Conditional direct effect of employee Mach on CWB*				
Low leader Mach	.29 (.07)	4.15 (.00)	.15	.42
Mean leader Mach	.27 (.05)	5.09 (.00)	.16	.37
High leader Mach	.25 (.07)	3.80 (.00)	.12	.38

First, the conditional indirect effects of employee Mach on stress and CWB via trust were not significant (see Table 3). Effects for stress ranged from .001 (boot s.e. = .02, boot 95 % CI [−.03, .05]) at low values of leader Mach (mean − 1 SD) to .02 (boot s.e. = .02, boot 95 % CI [−.00, .09]) at the mean value of leader Mach to .04 (boot s.e. = .03, boot 95 % CI [−.01, .14]) at high values of leader Mach (mean + 1 SD). Effects for CWB ranged from .001 (boot s.e. = .01, boot 95 % CI [−.02, .03]) at low values of leader Mach to .01 (boot s.e. = .01, boot 95 % CI [−.00, .06]) at the mean, and to .03 (boot s.e. = .02, boot 95 % CI [−.003, .10]) at high values of leader Mach.

**Fig. 2:**
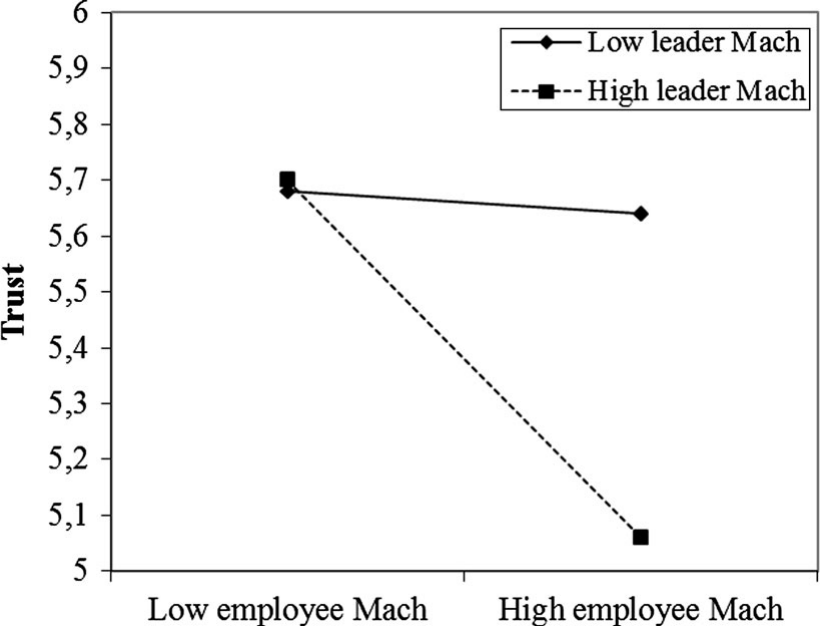
Interaction effect of leader and employee Mach for employee trust

Employee Mach did have a conditional direct effect on trust, that is, the relationship between employee Mach and trust was moderated by leader Mach (first stage moderation). This effect was −.01 (s.e. = .10, *t* = −.06, *p* = .95, 95 % CI [−.21, .20]) for low values of leader Mach (mean – 1 SD), −.17 (s.e. = .08, *t* = −2.24, *p* = .03, 95 % CI [−.33, −.02]) at the mean, and −.34 (s.e. = .09, *t* = −3.61, *p* = .00, 95 % CI [−.53, −.15]) for high leader Mach. Figure 2 shows the plots of the high versus low leader Mach regression lines. Employee Mach was only significantly (negatively) related to employee trust for leaders high on Mach. Results of single slope analyses confirmed that the slope for high leader Mach was significant whereas the one for low leader Mach was not. High employee Mach combined with high leader Mach thus came with the lowest levels of trust.

**Fig. 3:**
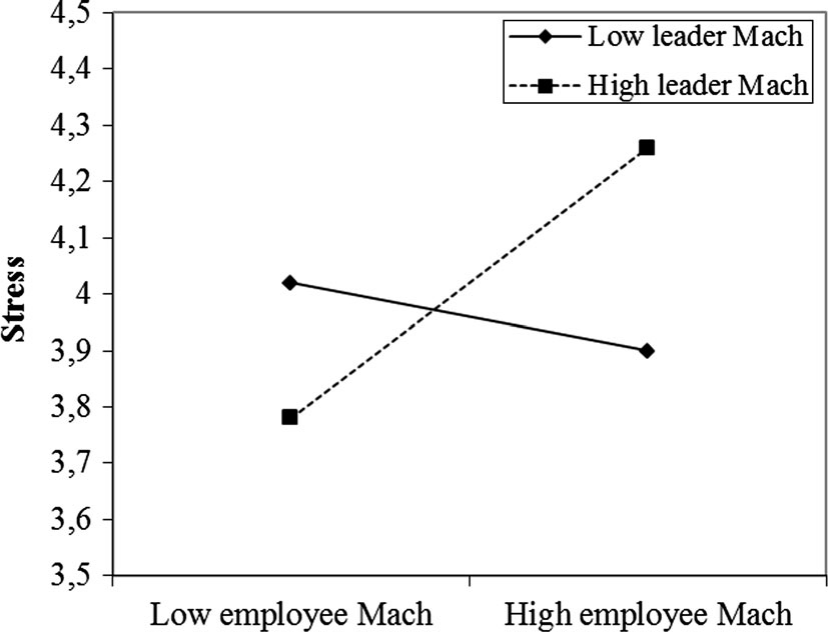
Interaction effect of leader and employee Mach for employee stress

Interestingly, we also found a conditional direct effect of employee Mach on stress (direct effect moderation). The effect was −.07 (s.e. = 13, *t* = −.60, *p* = .55, 95 % CI [−.32, .17]) at low leader Mach, .09 (s.e. = .10, *t* = .95, *p* = .35, 95 % CI [−.10, .28]) at the mean, and .25 (s.e. = .12, *t* = 2.15, *p* = .03, 95 % CI [.02, .49]) at high leader Mach. Figure 3 presents the plots of the regression lines for high versus low leader Mach. Employee Mach was only significantly (positively) related to employee stress when leader Mach was high. Single slope analyses confirmed that the slopes were only significant for high leader Mach and not for low leader Mach. Highest levels of stress were experienced in a situation in which high-Mach employees were combined with high-Mach leaders.

For CWB we found a significant main effect of employee Mach on CWB. The direct effect of employee Mach on CWB was not conditional but rather significant for low, mean, and high levels of leader Mach (mean – 1 SD: effect = .29, s.e. = .07, *t* = 4.15, *p* = .00, 95 % CI [.15, .42]; mean: effect = .27, s.e. = .07, *t* = 5.09, *p* = .00, 95 % CI [.16, .37]; mean + 1 SD: effect = .25, s.e. = .07, *t* = 3.80, *p* = .00, 95 % CI [.12, .38]).

As an additional analysis (and in line with Hypothesis 1), we conducted a mediation analysis without including leader Mach as a moderator using the PROCESS macro. With no moderator in the model, the indirect effect of employee Mach via trust was found to be significant for stress (effect = .05, boot s.e. = .03, boot 95 % CI [.01, .13] but not for CWB (effect = .03, boot s.e. = .02, boot 95 % CI [−.001, .08]).

## Discussion

As personality factors are relatively stable and cannot easily be changed but do affect how people interact at work, it is essential for organizations to understand the influence of employee as well as leader personality on employee performance and well-being, workplace interactions and (un)ethical behavior, and other important outcomes (e.g., Anderson et al. 2008; Barrick et al. 2002). Of particular concern is how those with ‘dark’ personality traits such as high Machiavellianism (Paulhus and Williams 2002) function while working with others due to the links that Mach has with making unethical choices and showing destructive behaviors in the workplace (e.g., Schepers 2003; Smith et al. 2009). Some studies focus on Mach as part of the “dark triad” (e.g., Rauthmann 2012; see Furnham et al. 2013), combining Mach, narcissism, and psychopathy in a single factor. However, extant research shows that the three constructs are only moderately correlated and should rather be investigated separately (e.g., Jones and Paulhus 2011; Paulhus and Williams 2002; Stellwagen 2011). The current study thus focused specifically on Machiavellianism and adds to the field in several respects.

First, our study extends the current perspective on Machiavellianism in organizations which usually investigates the effects of Machiavellianism in employees themselves (see Dahling et al. 2009) by also including the impact of Machiavellianism in leaders on relevant outcome variables. This adds to the few extant empirical studies on Machiavellian leadership (e.g., Deluga 2001), which so far have focused mainly on performance variables as outcomes. To our knowledge, work to date has not yet investigated the effects of Machiavellian leadership on disruptive variables such as employee stress or lack of trust. Interestingly, relationships with outcome variables were similar for Mach leadership and employee Mach: both employee Mach and leader Mach were significantly correlated with employees’ lack of trust and their engagement in unethical behavior (CWB). Machiavellianism thus seems to have the potential to have detrimental effects on employee attitudes and behavior no matter at which level it is present (employee or leader).

Employee Mach and leader Mach were also significantly and substantially correlated with each other (*r* = .48, *p* < .01). Given the conceptualization of Mach as a stable personality variable, selection effects might take place in the sense that Mach leaders might attract Mach employees, or Mach employees might attract Mach leaders thus leading to the risk of an accumulation of individuals high on Mach in teams. However, future work might also explore whether both trait and state like elements of Mach exist, and whether situational cues such as working for a high Mach leader may (at least to some extent) ‘trigger’ an increase in employees’ Mach orientation and behavior.

This study also adds Machiavellian leadership as an additional ‘style’ to the field of negative, destructive, and unethical leadership (e.g., Einarsen et al. 2007; Rosenthal and Pittinsky 2006; Tepper 2000). Even though research has shown that abusive supervision is negatively related to employee well-being and positively related to employee workplace deviance (see Tepper 2007), and that Machs are perceived (at times) to engage in abusive leader behavior (Kiazad et al. 2010), Machiavellianism also differs from abusive supervision. While the former is defined as a sustained display of hostile behavior, Machs only exhibit hostile behavior (e.g., intimidation) if they perceive doing so as instrumental to achieving their ends (e.g., Wilson et al. 1996). Machs are flexible and strongly goal driven. As Wilson et al. (1996, p. 295) note, it is also “part of the Machiavellian strategy to be genuinely cooperative, trustworthy, and so forth when it is advantageous.” Machiavellianism thus seems to be an important leader personality variable that organizations need to manage effectively in order to avoid negative reactions of employees. For example, the findings of Den Hartog and Belschak (2012) suggest that the negative effects of leader Mach on employee workplace deviance can be reduced by explicitly engaging in ethical leader behaviors.

Our study replicates previous findings on Machiavellianism in employees and corroborates the link of employee Mach with important organizational outcomes such as reduced trust, increased stress, and more unethical behavior (see also Dahling et al. 2009). As current empirical research on Mach is still sparse, this study adds to our overall understanding of how those with negative personality traits act in organizations in general and respond to different organizational settings (see also the limited but steadily growing research in the area of business ethics on this topic: Dahling et al. 2012; Ricks and Fraedrich 1999; Schepers 2003; Winter et al. 2004). Yet, we further specified the relationship between these variables by proposing a mediation model in which employee Mach was related to stress and CWB via employee lack of trust in the leader. While the data substantiated the hypothesized general mediation model for stress, we did not find support for a moderated mediation model proposing a conditional indirect effect of employee Mach on employee stress via trust. Rather, we found conditional direct effects of employee Mach and leader Mach on both employee trust in the supervisor and employee stress. Employees who were high in Mach exhibited decreased trust in their leader and increased stress when paired with Mach leaders.

Earlier work on Mach suggests that suspicious supervisors of Mach employees may see through their manipulative intentions and distrust them; they may then restrict the amount of information, authority, and control given to this subordinate, which creates an increased level of stress for the Mach employee (Gemmill and Heisler 1972). With a leader who is high on Mach, who has a negative worldview and expects the worst of others, and who tries to manipulate them, the tendency to withhold information and restrict employee autonomy is likely to be even stronger. The probability of maintaining a high level of flexibility to enact their own goals therefore decreases for employees of Mach leaders (McHoskey 1999). Yet, Mach employees highly appreciate flexible, high-autonomy situations which offer them room to maneuver (e.g., Belschak et al. 2015), and such a reduced sense of autonomy and control is therefore likely to result in an increase in stress and decrease of trust in the leader for these employees.

Additionally, Kiazad et al. (2010) argue that Mach biases leader behavior toward showing more hostility and negative behaviors and found that employees perceived Mach leaders as being more abusive. Abusive leadership, in turn, often stimulates a significant amount of stress (see Tepper 2007) and distrust (cf. Brown et al. 2005) in employees. Mach employees tend to be more sensitive to stress and feeling pressured due to their negative worldview (e.g., Dahling et al. 2009), and our results suggest they experience even more stress and less trust than non-Mach employees when paired with a controlling, hostile Mach leader. To our knowledge, this had not yet been investigated to date and creates a new line of research investigating interactions between Mach and other personality types (here, the other person’s Mach).

Somewhat unexpected, we did not find support for our hypotheses regarding employee CWB as we only found a direct effect of employee Mach and not an interactive effect. It might be possible that in reaction to the leader, employees choose to direct their negative, counterproductive behaviors more specifically at their distrusted Machiavellian leader rather than at the organization more broadly. Employees may not generally hold their organization responsible for their negative leader. It could thus be interesting to test an adapted version of our moderated mediation model in which employee Mach is related to trust in the leader which, in turn, is related to counterproductive behavior directed at the leader, with leader Mach acting as a first stage moderator. In addition, employee Mach might be linked to a (lack of) trust in the organization and, in turn, to CWB directed at the organization. Such alternative models would be in line with the target similarity model which argues that the foci of social exchanges (here, a negative exchange between the leader and the employee) need to be compatible, i.e., a Mach personality of the leader and a lack of trust toward the leader should lead to negative behavior targeting specifically the leader (see Lavelle et al. 2007).

Machiavellians’ manipulative, goal-directed, self-serving behavior can be linked to research on organizational politics, which is defined as (unsanctioned) self-interested influence attempts at the expense of organizational goals (e.g., Ferris et al. 1989). Studies in this area found that perceptions of such political behavior stimulate negative employee reactions like turnover intentions, CWB, and stress (Cropanzano et al. 1997; Randall et al. 1999). In organizations with Mach leaders, employees likely experience a high extent of organizational politics. In this sense, our findings about the (moderating) effects of leader Mach could be transferred to the field of organizational politics. A political organizational environment might be most detrimental for ‘dark’ employees (e.g., employees high on Mach) and exacerbate the dispositional negative tendencies of such employees. Our findings thus can add to the emerging literature on the interactive effects of organizational politics and employee personality (e.g., Witt et al. 2002), and this combination with organizational politics forms an interesting area for future research.

Finally, we add to the growing literature regarding leader–follower personality similarities and clashes (Antonioni and Park 2001; Bauer and Green 1996; Senger 1971; Turban and Jones 1988). Leader–follower fit has been an important topic in leadership research (e.g., Atwater and Dionne 2007). This study forms an empirical test of Atwater and Dionne’s (2007) leader–follower fit theory for a negative trait. While most studies focus on beneficial effects of leader–follower fit (e.g., Giberson et al. 2005), studies that argue for (and find) more beneficial effects for leader–follower complementarity are scarce (for an exception on dominance see Grant et al. 2011). Here, we did not find support for a fit model as a high Mach–Mach combination led to the most negative results on outcomes. This provides additional support for research arguing that leader–follower similarity is not always desirable and may have detrimental effects, and that, in addition to similarity, leader–follower complementarity also needs consideration when assessing fit. We also extend the discussion on leader–follower fit by focusing on a *negative* personality factor which, to date, has not received much attention in this stream of research.

### Practical Implications

From a practical perspective, this study offers several suggestions. The finding that both employee Mach and leader Mach were negatively linked to disruptive outcome variables suggests that Machiavellians might indeed be a group of individuals who need to be carefully managed if they enter the organization. One study suggests that transformational leadership is an effective style to lead Mach employees toward pro-organizational behaviors (Belschak et al. 2015), yet more research on this is needed.

The results of our study indicate that the management of high-Mach employees becomes even more crucial the moment that these employees attain a leader position. Here, these individuals not only present the risk of acting in ways detrimental to the organization itself, their employees also react in negative ways to such leaders, thus amplifying the negative organizational impact of Machiavellian leadership. Organizations might therefore be well-advised to carefully consider these negative effects and weigh the benefits and costs of promoting a high-Mach employee into a leadership position. On the one hand, Mach leaders are often perceived as charismatic and might “get things done” (Deluga 2001) and, if properly managed themselves, perhaps they can be directed in organizationally desirable ways (see Belschak et al. 2015). On the other hand, if *not* managed properly, these Mach leaders bear the risk of seriously damaging the organization.

Finally, our study suggests that combining Mach employees with Mach leaders is a situation that organizations should try to avoid. Such a toxic combination of leader–follower personality may provide a climate that can be damaging for both employee well-being and leader–follower interactions. Organizations should therefore be careful to assign Mach employees to non-Mach leaders whenever possible; under these conditions, our findings suggest, the non-Mach leader seems to buffer the negative effects of employee Mach. Further work on this buffering effect would be of interest.

### Limitations and Conclusions

Like most studies, this study suffers from a number of limitations. First, we aimed to broadly explore the negative effects of Mach in organizations by including outcome variables at the individual (stress), relational (trust), and organizational level (CWB). Yet, we investigated only a limited number of variables. It might be interesting to explore the consequences of employee Mach–leader Mach also for other outcome variables such as performance, turnover, or absenteeism. Are Mach employees and leaders in combination somehow still able to be productive and cope with the situation? It might be that for these personality types stress and a lack of trust ultimately do not translate into these undesirable final consequences even though the extant literature suggests that generally such performance and withdrawal variables are related to stress and trust.

Next, our outcome measures were measured as employee ratings. This bears the risk of introducing common method variance to part of our study (Hypothesis 1). Yet, the main contribution of our study lies in the multi-source part in which we investigate the effects of Machiavellian leadership on employee outcomes and in the interaction of employee Mach and leader Mach on employee reactions. These research questions (Hypothesis 2) have not been investigated to date and rely on multi-source data. Also, the findings of the data based on a common source are in line with existing research on this topic (see Dahling et al. 2009; Jones and Paulhus 2009).

Another limitation that needs to be acknowledged is the use of cross-sectional data to test a mediation model, which assumes that causal processes unfold over time. Even though such a method is frequently used in the field, it comes with the problem of potentially biased results (Maxwell and Cole 2007), and further longitudinal research is needed here. Yet, as mentioned above, the focal contribution of this study concerns the interaction between employee Mach and leader Mach which is not subject to these concerns.

Further, we used short versions of longer, validated scales as measures of our focal constructs which may be problematic as the shortened scales may measure different constructs than the longer, parent scales (Little et al. 1999). However, the shortened scales from our study have been successfully used in earlier studies providing some validity evidence of these specific measures (e.g., Belschak and Den Hartog 2009; Belschak et al. 2015; Den Hartog and Belschak 2012; Fox and Spector 1999).

Finally, we have only explored the reactions of *employees* to employee Mach–leader Mach interactions. Future research should also consider gathering responses from leaders to understand how Mach leaders may feel when working with Mach followers. Do they react in similar ways to employees, or, since they are higher on the hierarchy, does this affect them less? How do other coworkers feel when they are in the presence of this toxic Mach combination? Is their work affected, and do they engage in increased unethical behavior, too? Additionally, longitudinal studies should be carried out to understand the long-term consequences of working with the Mach personality. Do non-Mach employees become more Mach-like over time? How do employees and leaders cope with and solve such situations? Longitudinal research could strengthen conclusions and explore such related questions.

In conclusion, a contribution of this study lies in the exploration of the disruptive effects of Machiavellian leadership on employees in general and on Machiavellian employees in particular. This has not yet been addressed in research to date. Our findings suggest that Machiavellians should be managed carefully in order to avoid detrimental consequences for the organization. Clearly more research is needed on how to effectively manage this specific group of members of the organization.
